# Bioinspired Superdurable Pestle‐Loop Mechanical Interlocker with Tunable Peeling Force, Strong Shear Adhesion, and Low Noise

**DOI:** 10.1002/advs.201700787

**Published:** 2018-02-09

**Authors:** Junrong Jiao, Feilong Zhang, Tian Jiao, Zhen Gu, Shutao Wang

**Affiliations:** ^1^ CAS Key Laboratory of Bio‐inspired Materials and Interfacial Science CAS Center for Excellence in Nanoscience Technical Institute of Physics and Chemistry Chinese Academy of Sciences Beijing 100190 P. R. China; ^2^ University of Chinese Academy of Sciences Beijing 100049 P. R. China; ^3^ Beijing National Laboratory for Molecular Sciences Key Laboratory of Green Printing Institute of Chemistry Chinese Academy of Sciences Beijing 100190 P. R. China; ^4^ College of Mechanical Engineering Sichuan University of Science and Engineering Zigong 643000 P. R. China

**Keywords:** bioinspired structures, low noise, pestle‐loop mechanical interlockers, superdurability, tunable peeling forces

## Abstract

Velcro, the most typical hook‐loop interlocker, often suffers from undesirable deformation, breaking, and noise because of the structure of the hook. Inspired by the arrester system of dragonfly, a new mechanical interlocker with a nylon pestle instead of the traditional hook is developed. The pestle‐loop mechanical interlocker shows a tunable peeling force from 0.4 ± 0.14 to 6.5 ± 0.72 N and the shear adhesion force of pestle‐loop mechanical interlocker is about twice as much as that of velcro. The pestle tape can be separated and fastened with the loop tape up to 30 000 cycles while keeping the original adhesive force and the pestle structure. In comparison, only after 4000 cycles most hooks of the commercial velcro are deformed and even broken, completely losing their adhesive function and their hook structure. These experimental results are further supported by finite element simulitions—the base of pestle mainly bears the separation‐caused strain while the middle of hook does. Notably, the sound volume during the separation of pestle‐loop mechanical interlocker is merely 49 ± 7.4 dB, much lower than 70 ± 3.5 dB produced by the velcro.

Bioinspired materials have aroused intensive attention because of their unique properties, such as self‐cleaning effect from lotus,^1^ water‐collection capability from spider silk,^2^ superslippery,^3^ and directional water transport^4^ from *Nepenthes* and structure color from weevil.^5^ Gecko foot encourages another kind of amazing bioinspired study from the viewpoint of surface adhesion,^6^ together with other natural adhesive systems like plant,^7^ insect,^8^ tree frog,^9^ octopus,^10^ and mussel.^11^ The most popular mechanical interlocker, velcro^12^ (hook‐loop interlocker) inspired by the hooks of burdock, has been applied in many fields since it was invented, such as clothing, room apparatus, and medical bandages. However, the velcro often bother users by its deformation, break, and the separating noise. Therefore, it remains a great challenge to develop new generation of mechanical interlocker with high durability and less noise.

The “slim neck” of dragonfly can support the “big head” freely shaking for millions of times during feeding, pairing, and flying in the whole life. It was found that the pestle‐cone arrester system between its head and shoulders is capable to effectively protect the “slim neck” by constantly mechanical interlock and unlock.^13^ Inspired by the pestle‐cone interlocker of dragonfly, we develop a superdurable pestle‐loop mechanical interlocker (PLMI, including pestle tape and loop tape) with less separating noise, tunable peeling force, and strong shear adhesion (**Figure** **1**a,b). The pestle tape can be obtained through a simple heat treatment process from the hook tape (Figure 1c and Figure S1, Supporting Information). This study will bring new clues in designing next‐generation mechanical interlocker.

**Figure 1:**
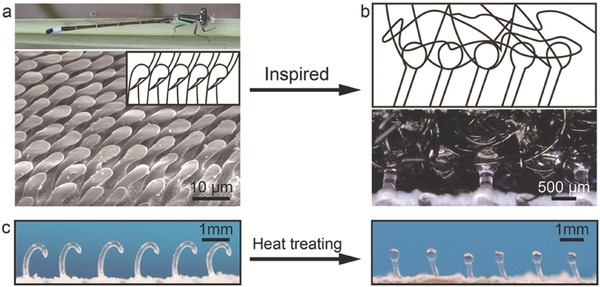
a) Optical images of dragonfly, images of pestle‐shaped cilia of arrester‐system (*Zygoptera*, *Ischnura elegans*). b) Mechanical interlocker model and optical images of pestle‐loop mechanical interlocker. c) Fabricated process of pestle tape.

At first, we compared the detachment forces from the single loop of the single pestle, the mushroom,^14^ and the hook along with increase of attachment–detachment cycles (**Figure** **2**a). The detachment force for the single pestle shows no apparent decreasing after 13 cycles, for single hook decreases to 27.1 ± 0.28% after 13 cycles and for mushroom sharply drops down to zero after eight cycles. The optical images also support this observation; after 13 cycles the pestle keeps its original shape. On the contrary, the hook has become straight from curved shape and the head of mushroom has separated from the stem after eight cycles. These results suggest that the pestle exhibits a higher durability than the hook and the mushroom.

**Figure 2:**
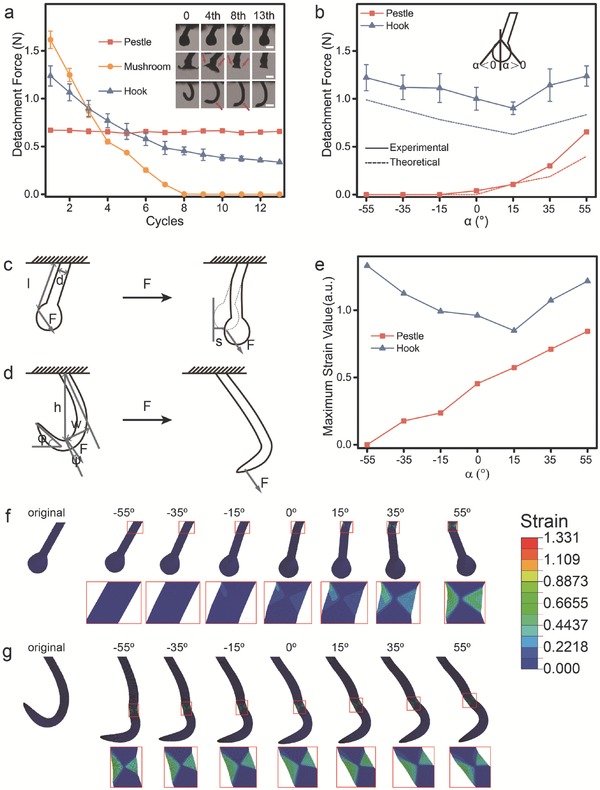
a) Detachment force of single pestle‐loop, mushroom‐loop, and hook‐loop after 1–13 cycles. Insert: Optical images of pestle, mushroom, and hook after 0, 4, 8, and 13 cycles, respectively. Scale bar, 300 µm (pestle, mushroom); 500 µm (hook). b) Detachment force of pestle‐loop, mushroom‐loop, and hook‐loop with different α of −55°, −35°, −15°, 0°, 15°, 35°, and 55°. c,d) Mechanics analyses of pestle‐loop and hook‐loop interlockers. e–g) At the detachment moment, maximum strain value and strain area of pestle and hook with the α from −55° to 55° simulated by Abaqus 6.10.

The detachment force for pestle is close to zero when the angle (α) between vertical and loop is less than 0°, and the detachment force gradually increases with the increasing of the α from 0° to 55°. The detachment force for hook decreases and then increases with the increasing of α and reaches the minimum at the α of 15° (Figure 2b).

We further attempt to understand the mechanical interlocker of single pestle and loop (Figure 2c). The detachment force *F* is related to the elastic tangential deflection *s*, the elastic modulus of materials *E*, the distance between final stress position and the base tape *l*, and the second moment of area section *I* by the conventional engineering small‐strain beam bending analysis, as Equation (1)

(1) $$F\;=\;\frac{2sEI}{l^{3}}\;\Rightarrow \;\frac{sE\pi d^{4}}{32l^{3}}$$

where *d* is the diameter of the stem of the pestle The *s* increases with the increasing of angle (α) from 0° to 55° (Figure S2, Supporting Information). And the *F* depends linearly on the *s* because the *E, l*, *d* are constants for the pestle‐loop interlocker.

For the hook‐loop interlocker, the force *F* is applied on the hook by the loop at an angle ψ to the direction of the shaft of hook, and the loop slips down from hook when the tip of hook rotated an angle (ϕ + ψ) (Figure 2d). The friction angle λ must be included in required angle (ϕ + ψ + λ) because of the significant friction between the hook and loop. It can be described as follows (Equation (2))^15^

(2) $$\frac{Fw}{EI}\left\{\frac{EI}{k}\;+\;h\;+\;\frac{w}{2}\right\}\;=\;\varphi \;+\;\psi \;+\;\lambda$$

where *k* is the rotational stiffness of hook root, *h* is the distance between the loop and the base tape, and *w* is the distance between the loop and the shaft of hook. The *F* is increased with the increasing of ψ because other parameters are fixed for the hook‐loop interlock. The shaft of hook has an angle of 18° ± 3.1° with the tape and therefore the ψ reaches the minimum at the α of 15°. The calculated detachment force (dashed line in Figure 2b) of the pestle‐loop and hook‐loop interlocker is consistent with the experimental data, respectively. The fixed parameters used in the above equations are listed in Table S1 (Supporting Information).

We exploit a modeling approach to understand the inherent dynamic mechanism of pestle‐loop and hook‐loop interlocker. With the increasing of α from −55° to 55°, the maximum strain value of single pestle increases (Figure 2e) and its main strain area locates at the base (Figure 2f). In comparison, the main strain area of single hook is at the middle of hook (Figure 2g) and its maximum strain value is higher than that of single pestle. In the simulation, the borders of pestle/hook and bottom tape are defined as the boundary. The definition has negligible effects to the simulation results of hook, but would greatly increase the strain values of pestle in the simulation. Actually, the stem of pestle was woven into the tape^12^ and the pestle would transfer the part strain to the base tape in the detachment. So the strains of pestles in actually are far less than the simulation strains. The simulation results further support the experimental results.

Next, we study the mechanical adhesion of PLMI by measuring max peeling force (*F*_MP_).^16^ The *F*_MP_ of PLMI and velcro all reach the maximum at the preloading angle of 45° (**Figure** **3**a). So we chose the preloading force with an angle of 45° for the following experiments. After the preloading (*F*_L_), *F*_MP_ of PLMI and velcro were tested from terminal I and terminal II (Figure S3, Supporting Information). The *F*_MP_ from terminal I are much larger than that from terminal II. Especially at peeling angle of 120°, 135°, and 150°, the *F*_MP_ from terminal I are about five times of that from terminal II (Figure 3b). The peeling angle also affects the *F*_MP_, the *F*_MP_ increases by three times as the peeling angle increases from 30° to 150° (Figure 3b). However, the velcro has no strong dependence on the preloading direction and peeling angle (Figure 3c). Unlike the peeling directionality of gecko foot and gecko‐inspired directional adhesion devices that depends on the asymmetric geometry,^17^ the peeling directionality of PLMI depends on the asymmetry of preloading in shear direction. The smallest unit of PLMI is two symmetrical pestles, which tilt to two opposite direction. The *F*_L_ increases the interlock probability and the α of pestles (tilt toward terminal I) and loops, but without obvious effect on the pestles tilted toward terminal II (Figure 3d). However, the *F*_L_ increases the interlock probability and the α of all hook‐loop interlockers at the same time ( 3d). The increasing of peeling angle can also increase the α of the pestle‐loop interlockers (Figure S4, Supporting Information). The *F*_MP_ of PLMI can be controlled from 0.4 ± 0.14 to 6.5 ± 0.7 N by tuning the peeling direction and peeling angle.

**Figure 3:**
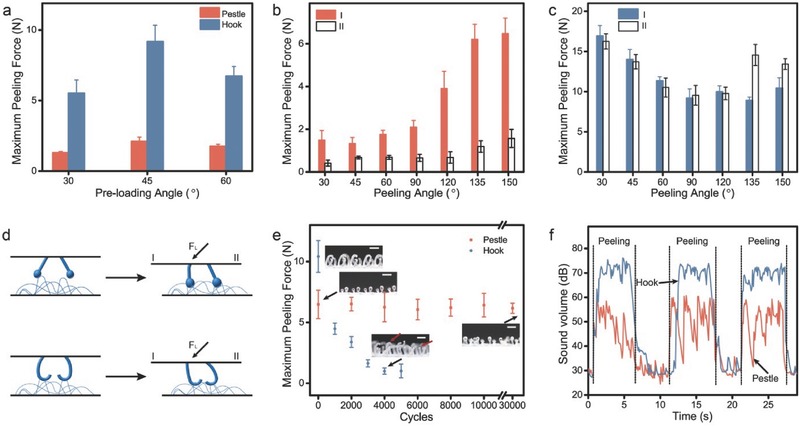
a) Maximum peeling forces (*F*_MP_) of pestle‐loop mechanical interlocker (PLMI) and velcro under the preloading with the angle of 30°, 45°, and 60°. b,c) *F*_MP_ of PLMI and velcro were tested from terminal I and II with peeling angle of 30°, 45°, 60°, 90°, 120°, 135°, and 150° and a peeling velocity of 0.33 mm s^−1^. d) The pestles and hooks after the preloading (*F*_L_). e) *F*_MP_ of PLMI after 0–30 000 cycles and the *F*_MP_ of velcro after 0–5000 cycles. Inset: The optical images of pestle tape after 0 and 30 000 cycles and the optical images of hook tape after 0 and 4000 cycles. The deformation and break of hooks were marked by red arrow. Scale bar, 1 mm. To demonstrate the pestles' durability well, we peeled the PLMI from terminal I with a detachment velocity of 50 mm s^−1^. f) Sound volume during the detachment of PLMI and velcro were detected with a detachment velocity of 50 mm s^−1^, peeling angle of 90°.

The pestle tape shows high durability even peeling from the terminal I with the peeling angle of 150°. The pestle tape allows for fastening and separating with loop tape over 30 000 cycles without reduction of max peeling force, the optical images also support this result; no obvious structural damage was observed (Figure 3e). According to Figure 2, the main deformation area at the base and low strain of pestle dramatically decrease the damage of pestle and sharply increase the durability of pestle. The max peeling forces for hook tape drop down to 43.0 ± 4.45% only after 1000 cycles because the hooks are straight or break.

The velcro always brings the irksome noise during the separation, while PLMI would not cause the noise. During the separation of PLMI, we observed obvious fluctuated sound volume and the average sound volume is 49 ± 7.4 dB. In contrast, velcro displays the continuous high noise of 70 ± 3.5 dB (Figure 3f). This difference may be caused by the strong mechanical interaction of hook‐loop and weak mechanical interaction of pestle‐loop. The strong mechanical interactions between hook and loop lead to the instantaneous resilience of loop, and cause the noise. Thanks to the tunable peeling force of PLMI, we can achieve the weak mechanical interactions between pestle and loops by adjusting the peeling direction and peeling angle. Just like across the strings, the weak mechanical interactions between pestle and loops cannot cause the strongly instantaneous resilience of loop.

In addition, the hook tape would nap the fabric, such as knitwear, sweater, etc. But the pestle cannot cause this undesirable damage. Normally, the undesirable contact of PLMI and wearing is nearly vertical. As displayed in **Figure** **4**a,b, the hooks would hook the loop and cause a large peeling force (8.22 ± 2.19 N), while the pestles are hard to interlock with the fiber of fabric under the vertical contact (less than 1 N). The optical images of knitwear before and after 5 and 100 cycles of attachment–detachment (Figure 4c) prove that pestle is wear‐friendly. The same results can be observed from the loop tape; the damage degree of loop tape with pestle tape after 1500 cycles is just similar to that with hook tape after 300 cycles (Figure 4d).

**Figure 4:**
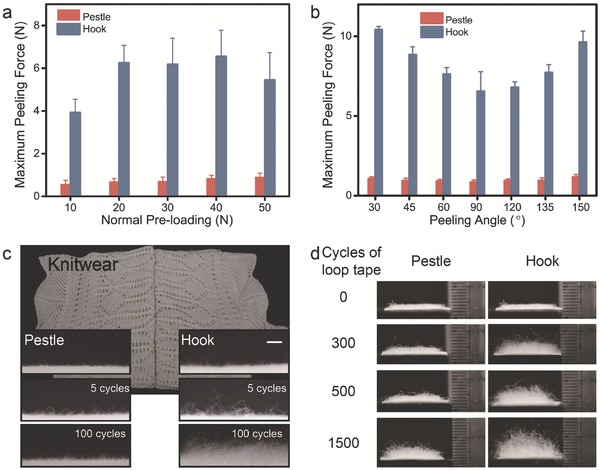
a) *F*_MP_ of PLMI and velcro were tested after the normal preloading of 10, 20, 30, 40, and 50 N, with the peeling angle of 90°. b) *F*_MP_ of PLMI and velcro were tested after a normal preloading (40 N) with peeling angle of 30°, 45°, 60°, 90°, 120°, 135°, and 150°. c) Optical images of knitwear (cotton fabric) before and after adhere to and detach from 5 and 100 cycles by hook and pestle tapes, respectively. Scale bar, 1 mm. d) Optical images of loop tape before and after test 0, 300, 500, and 1500 times. The PLMI and velcro were peeled with a detachment velocity of 0.33 mm s^−1^. The pestles had little damage to the loop tape. The damage of loop tape after test by pestle tape 1500 times was similar to that after test by hook tape 300 times.

The PLMI also exhibits strong shear adhesion force. As shown in **Figure** **5**a, the maximum shear adhesion force of PLMI (21.82 ± 0.32 N cm^−2^) is about twice of the velcro (10.77 ± 1.03 N cm^−2^). Figure 5b displays the shear detachment force of single pestle/hook with loop. There are 46 pestles/hooks cm^−2^. And accordingly, we can calculate the mechanical interlocking efficiency, 30.4% for PLMI and 16.7% for velcro. The pestle is easier to interlock with loop because the radius of pestle head (220 ± 7 µm) is less than the curvature radius of hook (350 ± 30 µm). The shear adhesion force of PLMI stabilizes at maximum for several seconds and then decreases sharply, while the shear adhesion force of velcro reaches its maximum and then decreases directly. Figure 5c,d shows the actual bearing weight of PLMI with the length of 8 cm is 17.3 kg, which is double the weight the velcro can bear. Even the PLMI can easily hang an adult with four 10 cm long tapes (Movie S1, Supporting Information).

**Figure 5:**
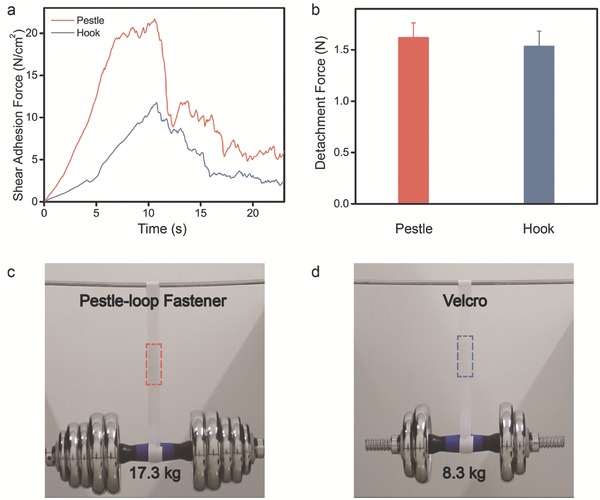
a) The pestle‐loop mechanical interlocker possesses about twice shear adhesion force of velcro. b) The shear detachment force from single loop of single pestle and hook. c,d) Optical images for showing the hold weight of PLMI and velcro.

In summary, we have presented a superdurable pestle‐loop mechanical interlocker with low separating noise, tunable peeling force, and strong shear adhesion inspired by the arrester system of dragonfly. The detachment force of single pestle‐loop interlocker is close to 0 when the α is less than 0°, and increase with the increasing of the α from 0° to 55°. Therefore, the pestle‐loop mechanical interlocker achieves tunable peeling force from 0.4 ± 0.14 to 6.5 ± 0.72 N and strong shear adhesion. The base of pestle undertakes the strain during the separation, which greatly improves the durability of pestle tape. The pestle tape can keep constant peeling force even after 30 000 cycles of separating and fastening between pestle and loop. In contrast, the hook tape nearly loses their function only after about 4000 cycles. Compared to the commercial velcro, the separating noise of pestle‐loop mechanical interlocker decreases by 21 dB. We believe this new type of pestle‐loop mechanical interlocker will greatly affect various fields, from fundamental research to practical applications.

## Experimental Section

*Materials*: The common commercial velcro was purchased from Twn‐G Ribbon Shanghai Co. Ltd. The effective width of hook and loop tapes is 1.5 cm.

*Preparation of Pestle Tape and Mushroom Tape*: The hook tape pass through the area, 1 mm over the alcohol lamp flame, with the speed of 20 mm s^−1^; the head of hook melted and formed a ball due to the surface tension, and the pestle tape was obtained. A clean glass slide (ultrasonic cleaning for 15 min in water, ethanol, and acetone, respectively) was placed on the hot stage at 300 °C for 5 min, then the pestle tape and a piece of metal (60 g) were placed on the slide for 5 s, and the slide was rapidly cooled using water. The ball of pestle was melted and spread out due to the gravity, and was molded by cooling. The mushroom was obtained. Figure S1 (Supporting Information) displayed the fabrication process of pestle and mushroom. The diameter of the stem of pestle, mushroom, and hook is from 201 to 205 µm, and the curvature radius of hook is from 323 to 383 µm. For pestle, the length of stem ranges from 500 to 610 µm, and the radius of ball ranges from 213 to 224 µm. For mushroom, the length of stem ranges from 500 to 575 µm, and the radius of head ranges from 330 to 350 µm.

*Optical Images of PLMI and Velcro*: The optical images and video shown in this paper were all taken by Canon camera (Canon 60D, Japan).

*Scanning Electron Microscopy (SEM)*: Before imaging, the sample surfaces were sputter‐coated with a layer of platinum. Images were obtained with a JEOL‐7500 scanning electron microscope (5 kV, 10 µA, JEOL, Tokyo, Japan).

*Measurement of Detachment Force, Peeling Force, and Shear Adhesion Force*: The detachment of single pestle, mushroom, and hook with loop was tested by a dynamometer (M5‐2, Mark‐10 Corporation) with a detachment velocity of 0.33 mm s^−1^. The dynamometer (M5‐50, Mark‐10 Corporation) was used to measure the maximum peeling force of PLMI and velcro. The triangular platforms were used to control preloading force direction and the peeling angle. The base angles of triangular platform were 30°, 45°, and 60° and the triangular platforms were named the first, second, and third platform according to the base angle, respectively. The test process for Figure 3B,C: the *F*_L_ (120 N) was applied and the PLMI and velcro were held on for 5 s by the second platform in the test; then the PLMI and velcro were taken from the second platform and fixed on the first, second, and third platforms, respectively; finally, the PLMI and velcro were peeled from preloading direction and it is opposite with a peeling velocity of 0.33 mm s^−1^, respectively. In order to maintain a constant peeling angle, the triangular platform at bottom was sliding. The max peeling force after *F*_LS_ was tested by dynamometer (M5‐50, Mark‐10 Corporation), and the peeling angle was achieved by the first, second, and third platforms. The shear adhesion forces were tested by dynamometer (M5‐50, Mark‐10 Corporation) with a detachment velocity of 0.83 mm s^−1^.

*Measurement of Sound Volume*: The noise volume was determined by precision pulse sound level meter (AWA5661‐3, Hangzhou Aihua Instruments Co. Ltd.). The noise volume of the first separation of PLMI and velcro was only determined with a peeling velocity of 120 mm s^−1^.

*Finite Element Simulation of Pestle, Hook with Loops*: For simplicity, 2D finite element models of single pestle and single hook were developed to simulate the dynamic effect of hook and pestle. The pestle and hook models contained 3449 and 6378 quadrate elements with very fine meshes in contact region between pestle/hook and loop, respectively. The final mesh density was determined through a series of convergence studies. Appropriate boundary conditions were used along the border of pestle/hook and bottom tape. The loop was simplified into a curve and sets as analytical rigid. The simulations were performed using Simulia Abaqus 6.10.

## Conflict of Interest

The authors declare no conflict of interest.

## Supporting information

Supplementary

Supplementary
