# Supplementary material for: Bioinspired Superdurable Pestle‐Loop Mechanical Interlocker with Tunable Peeling Force, Strong Shear Adhesion, and Low Noise
Source: Adv Sci (Weinh). 2018 Feb 9;5(4):1700787. doi: 10.1002/advs.201700787 (PMC5908517; doi:10.1002/advs.201700787)
Supplement: Supplementary file 1 — Supplementary [file ADVS-5-1700787-s002.pdf]

Copyright WILEY-VCH Verlag GmbH & Co. KGaA, 69469 Weinheim, Germany, 2018.

ADVANCED SCIENCE
Open Access

Supporting Information

for *Adv. Sci.,* DOI: 10.1002/advs.201700787

Bioinspired Superdurable Pestle-Loop Mechanical Interlocker with Tunable Peeling Force, Strong Shear Adhesion, and Low Noise

*Junrong Jiao, Feilong Zhang, Tian Jiao, Zhen Gu, and Shutao Wang**

Copyright WILEY-VCH Verlag GmbH & Co. KGaA, 69469 Weinheim, Germany, 2016.

Supporting Information

Bio-inspired superdurable pestle-loop mechanical inerlocker with tunable peeling force, strong shear adhesion and low noise

*Junrong Jiao, Feilong Zhang, Tian Jiao, Zhen Gu and Shutao Wang**

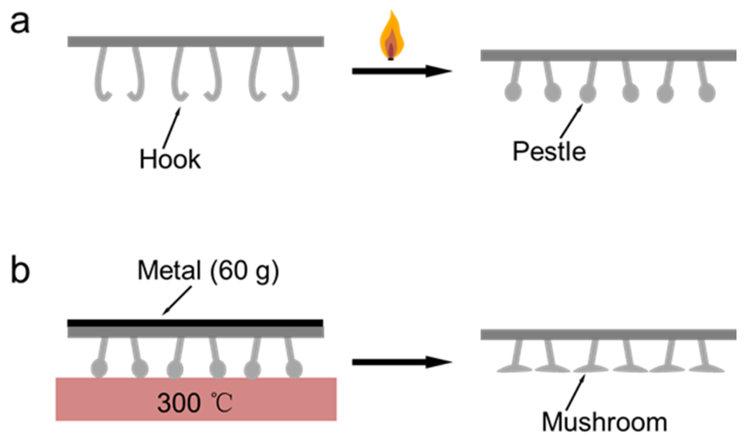

Figure S1 a) The hook tape pass through the area, over the alcohol lamp flame 1mm, with the speed of 20 mm s^{-1}; the head of hook melted and formed a ball due to the surface tension, and the pestle tape was obtained. b) Put the clean glass slide on the hot stage at 300 ℃ for 5 min, then put the pestle tape and a piece of metal (60 g) on the slide for 5 s. The ball of pestle melted and spread out due to the gravity, and was molded by cooling. Peeling the tape from the glass, and the mushroom was obtained.

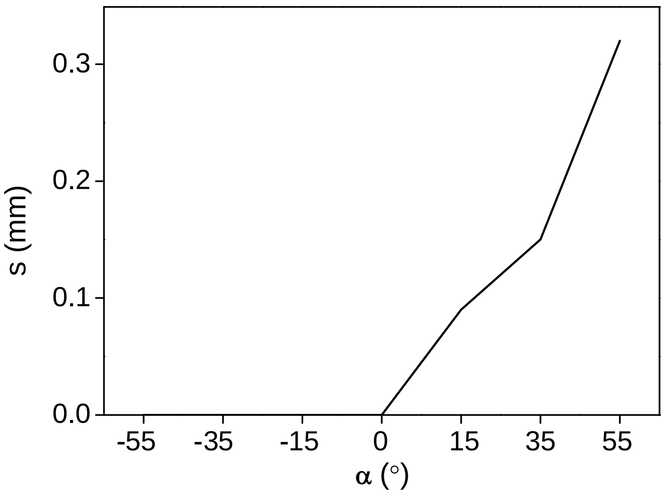

Figure S2. The numerical value of elastic tangential deflection *s* at the α of -55°, -35°, -15°, 0°, 15°, 35°, 55°. In the formula $F = \frac{2sEI}{l^3} \Rightarrow \frac{sE\pi d^4}{32l^3}$, the *E*,*d*,*l* are fixed for the pestle-loop interlock. The detachment force *F* of single pestle-loop interlocker is proportional to the elastic tangential deflection *s*.

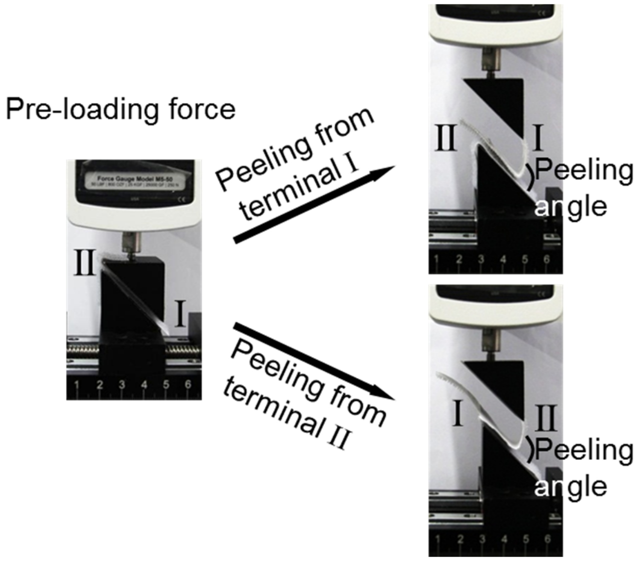

Figure S3. A pre-loading force (F_L) was applied on the PLMI and velcro, which can be divided into a vertical and a shear direction force. Unloading the pre-loading, peeled the PLMI and velcro from pre-loading direction and its opposite, respectively.

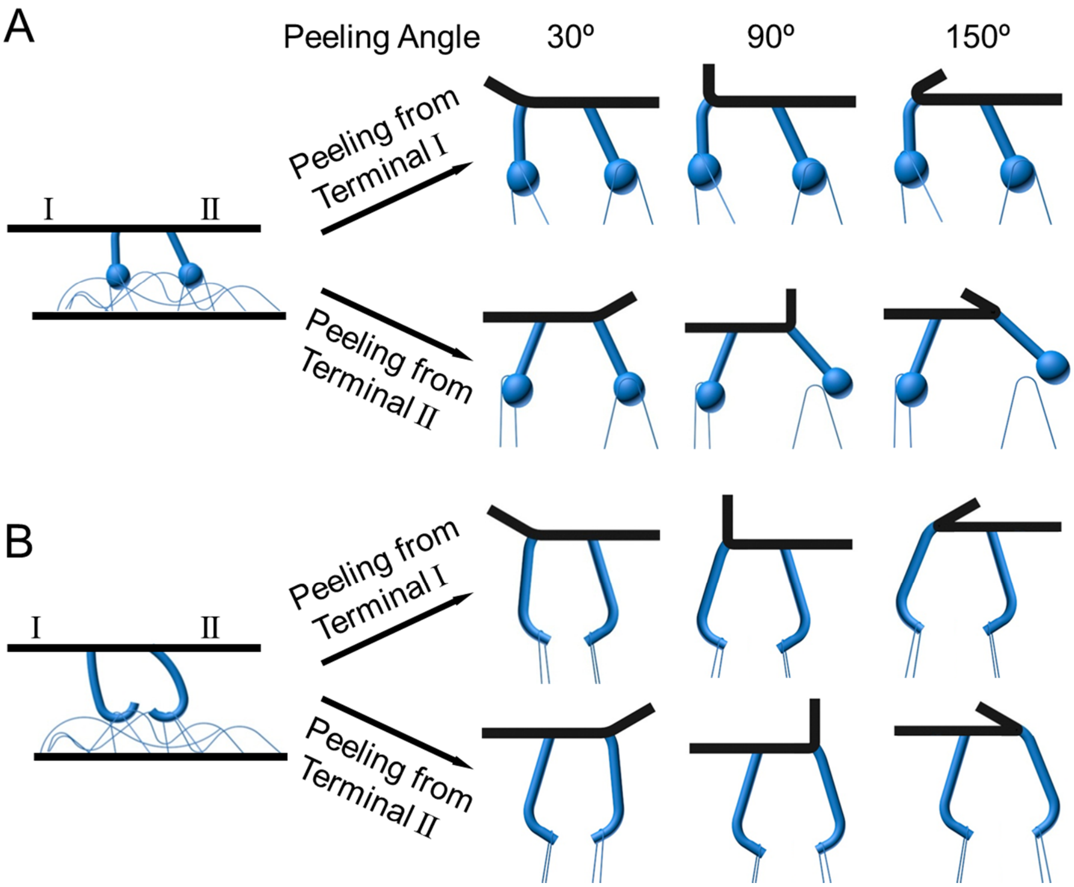

Figure S4 Schematic illustration of directional of PLMI. a-b) After the pre-loading, peeling the PLMI and velcro from terminal I and II with peeling angle of 30, 90 and 150 º, respectively. The increasing of peeling angle increase the α of pestle-loop and hook-loop interlockers.

Table S1. The numerical values of the fixed parameters in the Equation 1 and Equation 2.

E	d	l	k	h	w	φ	λ
2.8 GPa	0.203 mm	0.72 mm	Infinity	1.26 mm	0.52 mm	50º	72º

Movie S1

The PLMI can be easy to hang an adult of 56.4 kg, while the vlecro cannot. There are 4 PLMIs and velcros of 10 cm long. The PLMI and velcro are marked by the orange square.
